# Supplementary material for: Effect of sacubitril/valsartan on lipid metabolism in patients with chronic kidney disease combined with chronic heart failure: a retrospective study
Source: Lipids Health Dis. 2024 Feb 28;23:63. doi: 10.1186/s12944-024-02051-x (PMC10900560; doi:10.1186/s12944-024-02051-x)
Supplement: Supplementary file 1 — Supplementary Material 1. [file 12944_2024_2051_MOESM1_ESM.pdf]

17805922_ManzhiLi.docx

WORD COUNT 4792
TIME SUBMITTED 03-FEB-2024 04:37PM
PAPER ID 106642598

Effect of Sacubitril/Valsartan on lipid metabolism in patients with chronic kidney disease combined with chronic heart failure: a retrospective study

Abstract

Background and Objective

Dyslipidemia is significantly more common in those with concurrent chronic kidney disease (CKD) and chronic heart failure (CHF). Sacubitril/Valsartan has showcased its influence on both cardiac and renal functions, extending its influence to the modulation of lipid metabolism pathways. This study aims to examine how Sacubitril/Valsartan affects lipid metabolism within the context of CKD and CHF.

Methods

This study adopted a retrospective design, focusing on a single center and involving participants who were subjected to treatment with Sacubitril/Valsartan and Valsartan. The investigation assessed the treatment duration, with a particular emphasis on recording blood lipid indicators, including triglyceride (TG), total cholesterol (TC), low-density lipoprotein cholesterol (LDL-C), high-density lipoprotein cholesterol (HDL-C), apolipoprotein A (ApoA), and apolipoprotein B (ApoB). Furthermore, cardiac and renal functions, blood pressure, potassium levels, and other factors influencing the blood lipids were analyzed in both groups at identical time points.

Results

After 16 weeks of observation, the Sacubitril/Valsartan group exhibited lower TG levels compared to the Valsartan group. Noteworthy was the fact that individuals undergoing

Sacubitril/Valsartan treatment experienced an average reduction of 0.84 mmol/L in TG levels, in stark contrast to the control group, which registered a decline of 0.27 mmol/L (P < 0.001). The Sacubitril/Valsartan group exhibited elevated levels of HDL-C and ApoA in comparison to the Valsartan group (P_{HDL-C} = 0.023, P_{ApoA} = 0.030). While TC, LDL-C, and ApoB decreased compared to baseline, the differences between groups were not statistical significance. Regarding cardiac indicators, there was an observed enhancement in the left ventricular ejection fraction (LVEF) within the Sacubitril/Valsartan group when compared to the baseline, and it was noticeably higher than that of the Valsartan group. Spearman correlation analysis and multifactorial logistic regression analysis revealed that medication, body mass index, and glycated hemoglobin had a direct influencing effect on TG levels.

Conclusion

Sacubitril/Valsartan demonstrated improvements in lipid metabolism and cardiac indicators in patients with CKD and CHF. Specifically, it presented promising benefits in reducing TG levels. In addition, both BMI and HbA1c emerged as influential factors contributing to alterations in TG levels, independent of the administration of Sacubitril/Valsartan.

Keywords

Sacubitril/Valsartan, lipids, chronic kidney disease, chronic heart failure.

Introduction

Globally, approximately 15-20% of adults endure the presence of chronic kidney disease (CKD).

Extended retention of fluid induces increased cardiac stress and activates the renin-angiotensin-aldosterone system (RAAS)[1]. Chronic sympathetic stimulation can result in compromised cardiomyocyte function, diminished ventricular contractility, and the onset of cardiac insufficiency[2]. In individuals with CKD, cardiovascular disease (CVD) has emerged as the primary factor of adverse long-term outcomes[3]. Those with CKD and CVD commonly experience dyslipidemia. Substantiated by pertinent research, dyslipidemia independently contributes to the risk of CKD and CVD, exerting harm on the kidneys through systemic inflammatory responses, vascular injury, and oxidative stress[4]. Moreover, individuals experiencing hyperlipidemia face a cardiovascular disease risk twice as high as that of the general population. The chronic impact of lipid overload on the structural composition and function of the heart may contribute to initiation and advancement of chronic heart failure (CHF). Excessive lipid levels exert influence on both renal and cardiac systems, reciprocally influencing each other. Therefore, proactive management of lipid levels proves crucial for slowing disease progression and enhancing the prognosis for individuals with concurrent CKD and CHF.

Prior investigations have demonstrated the potential of statins in mitigating atherosclerotic risk among individuals grappling with both CKD and CVD. However, their use is marred by adverse effects such as rhabdomyolysis and hepatic insufficiency, with limited discernible benefits for patients undergoing dialysis therapy[5]. Emerging lipid-lowering interventions, such as proprotein convertase subtilisin/kexin 9 (PCSK9) inhibitors, may present side effects at the injection site, including allergies and muscle cramps[6]. The American Heart Association guidelines recommended utilizing omega-

3 fatty acids in individuals with heart failure (HF) to mitigate the likelihood of hospitalization and mortality, particularly in those categorized within New York Heart Association (NYHA) classes II-IV[7].

However, findings from the VITAL Rhythm study suggest that omega-3 fatty acids might contribute to an elevated risk of atrial fibrillation while concurrently exhibiting antiplatelet properties[8, 9]. Regular monitoring for bleeding risks is advised when using them alongside anticoagulants or antiplatelet agents. Therefore, the pursuit of secure and efficacious lipid-regulating strategies remains a pivotal focus for disease management and enhancing patient prognoses.

Origins of the natriuretic peptide (NP) family, including atrial natriuretic peptide (ANP) and brain natriuretic peptide (BNP), are found in atrial and ventricular myocytes. Natriuretic peptides (NPs), as elucidated by numerous studies, assume the functions of inducing vasodilation, promoting natriuresis, and inhibiting both the RAAS and the sympathetic nervous system[10-12]. The functional scope of NPs has been broadened by the presence of natriuretic peptide receptor (NPR) in human adipose tissue [13]. Galitzky et al. discovered a sustained lipolytic action of ANP through intravenous infusion into healthy and obese subjects, independent of the sympathetic nervous system[14]. In addition, Sbaraini da Silva et al. demonstrated a decrease in NP content among individuals with obesity[15]. Mice infused with BNP exhibited elevated expression of markers associated with energy expenditure, oxygen consumption, and brown adipose tissue compared to the non-BNP-infused group[16], indicating a close correlation between BNP and adipose tissue metabolism.

The adipocyte membrane's NPR and NP form a binding contact, instigating the activation of the cyclic guanosine monophosphate (cGMP)-protein kinase G (PKG) pathway. Consequently, this cascade facilitates hormone-sensitive lipase (HSL) phosphorylation, culminating in the hydrolysis of triglycerides and the generation of glycerol and non-esterified fatty acids[17].

Sacubitril/Valsartan, the novel inhibitor targeting angiotensin receptors and neprilysin, consists of sacubitril and Valsartan in a balanced 1:1 ratio[18]. Valsartan, through the inhibition of the angiotensin II (AngII) receptor, imparts therapeutic benefits, including antihypertensive effects, proteinuria reduction, and alleviation of cardiac load. Moreover, sacubitril serves as an enkephalinase inhibitor, impeding the breakdown of NPs and augmenting NP content. According to recent research, the NP route that promotes lipolysis is responsible for the enhancement of lipid levels among individuals with heart failure with preserved ejection fraction (HFpEF) when taking Sacubitril/Valsartan[19, 20].

One common risk factor for both CKD and CVD is dyslipidemia. Nevertheless, the current publications lack comprehensive exploration of the influence of Sacubitril/Valsartan on lipids within the CKD and CHF population. Therefore, the principal objective of this investigation was to discern the influence of Sacubitril/Valsartan on lipid levels among individuals with both CKD and CHF, with secondary objectives encompassing an evaluation of its effects on cardiac and renal function as well as blood pressure. The study hoped to contribute valuable insights into lipid management strategies for this specific population.

Subjects and Methods

1.1 Study methodology

This retrospective study focused on 212 individuals with CKD and CHF from a single center's sample. From January 2019 to November 2022, individuals within this patient demographic were admitted to the Affiliated Hospital of Xuzhou Medical University. The study included patients receiving Valsartan or Sacubitril/Valsartan treatment, and comprehensive clinical data was meticulously recorded utilizing an electronic case system. The Xuzhou Medical University Hospital's Ethics Committee granted the study approval (XYFY2023-KL142-02).

1.2 Patient selection

The specified inclusion criteria were as follows: manifestations and indications of HF, such as exertional dyspnea, nocturnal paroxysmal dyspnea, telangiectasia, ankle edema, N-terminal pro-brain natriuretic peptide (NT-proBNP) levels exceeding 400 pg/mL, NYHA class II - IV, estimated glomerular filtration rate (eGFR) less than 60 mL/min/1.73 m^2, abnormal urinary routine or renal imaging or pathology persisting for $\geq$ 3 months, age $\geq$ 18 years.

Exclusion criteria encompassed: a history of peritoneal dialysis, hemodialysis and kidney transplantation, notable bilateral renal artery stenosis, severe hepatic impairment, biliary cirrhosis, or cholestasis, systolic blood pressure (SBP) < 100 mmHg, potassium content > 5.5 mmol/L, history of stroke or acute coronary syndrome within 3 months before treatment, such as cardiac surgery or percutaneous coronary interventions (PCI), tumor-related diseases and definite drug-related renal damage,

familial hypercholesterolemia, history of angioedema, use of PCSK9 inhibitors, poor adherence, incomplete clinical data, loss of visits, intolerance of the side effects of the drug use and interruptions.

Throughout the medication period, patients in both groups adhered to a low-salt and low-fat diet, and the prescription of conventional medications such as beta-blockers, aldosterone antagonists and diuretics were determined by clinicians. Commencing at 25 mg twice daily, the initial dose of Sacubitril/Valsartan could be adjusted every two to four weeks. The dosage modifications were contingent upon the patient's tolerance levels related to blood pressure, heart rate, and symptoms. Generally, the maximum prescribed dose did not exceed 200 mg twice daily. The initial dose of Valsartan was 40 mg once daily, titrated according to guideline recommendations, without surpassing 160 mg twice daily.

1.3 Observation indicators and study objective

Baseline information and hematological indicators were collected both before and after 16 weeks of treatment. The utilization of antihypertensive drugs, statins, insulin, and other medications during the treatment period was meticulously documented through the electronic medical record system. Measurements of triglyceride (TG), total cholesterol (TC), low-density lipoprotein cholesterol (LDL-C), high-density lipoprotein cholesterol (HDL-C), apolipoprotein A (ApoA), apolipoprotein B (ApoB), serum creatinine, eGFR, cystatin C, urea, uric acid, fasting blood glucose (FBG), blood potassium, NT-proBNP, high-sensitive troponin T and hemoglobin A1c (HbA1c), were obtained from patients using an automated biochemical analyzer (Roche, Switzerland).

Additionally, the color Doppler ultrasound imager (Philips, Netherlands) was utilized to get the following measurements: left atrial diameter (LAD), left ventricular end-diastolic diameter (LVEDD), and left ventricular ejection fraction (LVEF).

Characterized by structural or functional abnormalities lasting over 3 months, chronic kidney disease (CKD) is identified. CHF is defined as abnormal systolic or diastolic function of the ventricles. The following criteria have been set by the Affiliated Hospital of Xuzhou Medical University to define normal or suitable blood lipid levels: TC $<$ 5.18 mmol/L, TG $<$ 1.70 mmol/L, HDL-C $\geqslant$ 1.04 mmol/L, LDL-C $<$ 3.37 mmol/L, ApoA $>$ 1.00 mmol/L, ApoB $<$ 1.14 mmol/L. The eGFR was calculated employing a four-variable equation outlined in 2006[21].

This study's primary goals were to assess changes in lipid indices within the two groups post-treatment, and to analyze factors influencing these variations in blood lipids. The secondary aim involved comparing alterations in cardiac and renal function indices, blood pressure, and blood potassium subsequent to medication.

1.4 Statistical methods

In this study, ten eligible patients were randomly chosen in each group, focusing on the change of TG levels in 16 weeks as the primary outcome in accordance with pertinent literature. The TG level in the Sacubitril/Valsartan group measured 1.86 ± 1.16 mmol/L, while in the Valsartan group, it registered at 1.36 ± 0.53 mmol/L. The study utilized PASS 15.0 software, adopting a 1:1 ratio for sample size, employing a one-sided α of 0.05, and achieving a test efficiency of 80%. The minimum required sample size was determined to be 84 cases in each group. To accommodate a potential loss to follow-up

of up to 20%, a minimum of 101 cases was included in each of the two groups. In total, there were 212 participants included in the study.Sacubitril/Valsartan served as the observation group and Valsartan was employed as the control group.

SPSS 26.0 software was used to analyze the data. For normally distributed quantitative data, mean and standard deviation were utilized to represent the values. Intergroup comparisons were conducted using the independent sample t test, while intragroup comparisons employed the paired samples t test. Non-normally distributed data were expressed using the median (quartile), with intragroup comparisons assessed through the Wilcoxon rank sum test and intergroup comparisons through the Mann-Whitney U test. Intergroup comparisons for categorical count data were conducted using chi-square tests, which were presented as the percentage of cases. The factors influencing TG decrease were examined using Spearman correlation analysis, multicollinearity testing and multiple linear regression analysis. At $P < 0.05$, statistical significance was taken into account.

2 Results

2.1 Baseline characteristics of the study participants

The study comprised 212 patients, with 106 assigned to the Sacubitril/Valsartan group and the remaining 106 to the Valsartan group. Medical histories and baseline data were comparable in both groups. The comparability of the clinical indicators was demonstrated by the lack of significant variations in intergroup lipid levels, cardiac and renal function, blood pressure and blood potassium (Table 1, Line 730).

2.2 Changes in blood lipid levels before and after treatment in both groups

After 16 weeks of therapy, the TG in the Sacubitril/Valsartan group exhibited a noteworthy reduction to 1.13 (0.84, 1.55) mmol/L ($P < 0.001$). In parallel, the TG levels in the Valsartan group also experienced a declined to 1.47 (1.15, 2.01) mmol/L ($P <$ 0.001). A comparative analysis of the magnitude of TG alteration during the treatment duration revealed a statistically distinction between groups. As depicted in Fig. 1, the Sacubitril/Valsartan group exhibited a change of 0.84 (0.40, 1.14) mmol/L, surpassing the Valsartan group's 0.27 (-0.11, 0.69) mmol/L ($P < 0.001$).

Throughout the treatment period, a significant reduction in TC was observed in the Sacubitril/Valsartan group, reaching 3.68 ± 1.12 mmol/L ($P < 0.001$). The Valsartan group exhibited a decrease in TC to 3.96 ± 1.36 mmol/L ($P < 0.001$). The intergroup disparities in TC post-treatment did not attain statistical significance ($P = 0.096$) despite these decreases. Comparing the post-treatment LDL-C levels with their respective pre-treatment values, significant differences were observed (2.05 ± 0.90 mmol/L in the observation group versus 2.17 ± 1.03 mmol/L in the control group, $P < 0.001$). Similarly, ApoB levels also displayed statistical significance (0.75 ± 0.26 mmol/L in Sacubitril/Valsartan group versus 0.82 ± 0.28 mmol/L in Valsartan group, $P_{\text{Sacubitril/Valsartan}} < 0.001$, $P_{\text{Valsartan}} = 0.007$). However, post-treatment comparisons between the two groups revealed no noteworthy differences in LDL-C and ApoB levels.

Following therapy, the Sacubitril/Valsartan group exhibited a mean HDL-C value of 1.24 ± 0.33 mmol/L, while the Valsartan group showed a value of 1.13 ± 0.34 mmol/L ($P_{\text{Sacubitril/Valsartan}} < 0.001$, $P_{\text{Valsartan}} = 0.881$). ApoA levels were elevated in both groups compared to pre-treatment ($P_{\text{Sacubitril/Valsartan}} < 0.001$, $P_{\text{Valsartan}} = 0.474$). After 16 weeks

of therapy, the Sacubitril/Valsartan group exhibited elevated levels of HDL-C and ApoA, as indicated by intergroup analysis ($P_{HDL-C} = 0.023$, $P_{ApoA} = 0.030$, Table 2, Fig. 2).

2.3 Changes in cardiac indexes before and after treatment

After treatment, the Sacubitril/Valsartan group showed a substantial improvement in LVEF and a noteworthy reduction in LVEDD, LAD, and NT-proBNP. In the control group, there were discernible reductions in both LVEDD and NT-proBNP, while no notable changes in LVEF and LAD were identified. After a 16-week treatment, the Sacubitril/Valsartan group exhibited the LVEF of 56.00 ± 6.71 mmol/L, surpassing that of the Valsartan group ($P < 0.001$). Additionally, the observation group exhibited a reduction in LAD to 40.39 ± 5.55 mmol/L, contrasting with the Valsartan group ($P = 0.001$). Both LVEDD and NT-proBNP exhibited no significant alterations in intergroup comparisons after treatment (Table 3).

Regarding the enhancement of cardiac function, the comprehensive efficacy rate reached 59.4% in the observation group post-treatment (contrasting with 38.7% in the control group), showcasing a discernible distinction between the two cohorts ($P = 0.003$, Fig. 3).

2.4 Changes in renal function before and after treatment

The Sacubitril/Valsartan group exhibited no obvious fluctuations in eGFR, blood creatinine, and uric acid after treatment ($P > 0.05$). Conversely, blood creatinine increased to 129.50 (106.00, 177.50) umol/L and eGFR decreased to 42.90 (29.93, 55.21) mL/min/1.73m^2 compared to the pre-treatment phase in the control group, which

had statistically significant differences ($P_{blood\ creatinine} < 0.001$, $P_{eGFR} = 0.001$). After treatment, no notable differences were observed about eGFR, blood creatinine, and uric acid in intergroup comparisons ($P > 0.05$), as indicated by the results presented in Table 4.

2.5 Variations in blood pressure and serum potassium levels at baseline and 16weeks

Over the course of 16 weeks, the Sacubitril/Valsartan group's systolic blood pressure (SBP) dropped from 146.35 ± 20.19 mmHg to 130.94 ± 22.87 mmHg ($P < 0.001$), a significant drop in intergroup comparisons ($P < 0.001$). Diastolic blood pressure (DBP) and potassium levels before and after therapy did not differ statistically significantly in intragroup or intergroup comparisons ($P > 0.05$), as presented in Table 5.

2.6 Analysis of factors affecting the amount of TG reduction

In order to investigate the factors affecting the extent of TG change, Spearman correlation analysis was conducted on the dataset of 212 patients. The reduction in TG was considered the outcome variable, and baseline data served as the independent variable. The analysis unveiled positive correlations between BMI and HbA1c with TG reduction [Spearman's rank correlation coefficient (r_s) 0.391 and 0.233, respectively]. Furthermore, patients undergoing Sacubitril/Valsartan treatment demonstrated a more substantial decrease in TG (r_s=0.343, $P < 0.001$), as depicted in Table 6 and illustrated in Fig. 4-5. To address confounding factors, a multicollinearity test was performed for BMI, HbA1c, and group, with all variance inflation factors found to be < 5, signifying an absence of collinearity. Moreover, a multiple linear regression model was formulated,

with group, BMI, and HbA1c as independent variables and the amount of TG reduction as the dependent variable. The results revealed that, in addition to the impact of BMI and HbA1c, the group emerged as a significant factor influencing the degree of TG reduction. In essence, the Sacubitril/Valsartan group exhibited a noteworthy impact on TG reduction post-treatment ($P < 0.001$, Table 7).

3 Discussion

In this study, individuals diagnosed with CKD and CHF, exhibiting an eGFR below 60 mL/min/1.73 m^2, were selected as participants. The objective revolved around scrutinizing the impact of Sacubitril/Valsartan in contrast to Valsartan on serum lipids, as well as cardiac and renal function indices. The overarching aim was to enhance understanding of Sacubitril/Valsartan's role in lipid metabolism.When compared between groups, subjects who received Sacubitril/Valsartan exhibited a substantial reduction in TG levels after 16 weeks of treatment. Apart from Sacubitril/Valsartan, both BMI and HbA1c have emerged as clinical factors influencing TG levels. Within the Sacubitril/Valsartan group, elevated levels in HDL-C, ApoA, and LVEF were evident in contrast to the Valsartan group. Conversely, TC, LDL-C, ApoB, and LAD displayed a decrease in the Sacubitril/Valsartan group. Notably, no appreciable disparity in renal function was discernible between the two groups.

Lipid abnormalities among CKD patients encompass hypertriglyceridemia, elevated LDL-C, ApoB accumulation, diminished HDL-C, lowered ApoA, and elevated lipoprotein a concentration[22, 23]. CKD individuals exhibit reduced enzyme activity of lecithin cholesterol acyltransferase, lipid accrual, and endothelial impairment,

accompanied by concurrent inflammatory and oxidative stress responses. Messow et al.'s meta-analysis, which incorporated 13 studies examining statin-treated CKD, revealed an escalation in cardiovascular risk with the progression of CKD stages[23]. Ho et al.'s cohort study found that fibrates may not effectively reduce cardiovascular risk[24]. This study focused on elucidating the influence of Sacubitril/Valsartan on lipid metabolism in individuals with CKD and CVD, intending to offer insights for future research.

In conventional wisdom, elevated blood lipid levels are commonly associated with advancing age. A comprehensive cohort study disclosed that, aside from age, gender differences were correlated with lipid levels. As males age, there was a discernible deceleration in the rate of alterations observed in TC, TG, and LDL-C, peaking before 40 years, while females experienced the most significant lipid level changes between ages 40-49, potentially attributed to the gradual decline in estrogen levels during the perimenopausal phase. Consequently, it is imperative for men to adopt suitable lipid management measures before reaching 40, and women should focus on such measures during the age range of 40-49[25, 26]. The average age of participants in this study was 69 years, with a predominant male representation. Following a 16-week treatment, no significant correlation emerged between the decrease in TG and age or gender. This lack of correlation could be attributed to diminishing or reversing differences in blood lipid levels associated with advancing age[27].

Barman et al.'s single-center retrospective study found that Sacubitril/Valsartan improved blood lipid levels, and its efficacy remained unaffected by statins[28]. In

patients with HFpEF, the outcomes of prospective trial showed a reduction in TG, an increase in HDL-C, and a slight rise in LDL-C[19]. The perspectives outlined above closely align with the findings of this study, with the exception of variations in LDL-C alterations. Given that the participants in this study presented with CKD in conjunction with CVD, the interplay between the heart and kidneys, along with the impact of NP mechanisms of action, could be responsible for the observed decline in LDL-C levels.

Over the course of 16 weeks of observation, alterations in TG levels consequent to Sacubitril/Valsartan treatment may be attributed to its inhibition of enkephalinase, preventing the decomposition of NPs. NPs are essential for fat oxidation, promoting energy expenditure in brown adipose tissue, and enabling lipid mobilization within white adipose tissue[29, 30]. NPs bind to receptors on the adipocyte membrane, activating the guanylyl cyclase A/B (Gc-A/B) through the cGMP/PKG pathway, known as the Gc-A/B/cGMP/PKG pathway[17]. Wang's research revealed a favorable correlation between ANP and HDL-C levels[31], which was consistent with the elevated HDL-C levels observed in this study. Diminished levels of ApoA, a component of HDL, were linked to an unfavorable prognosis in individuals with CHF[32]. The study found that Sacubitril/Valsartan increased ApoA content, suggesting a potential beneficial impact on HF patients' long-term prognosis.

Previous research have indicated low levels of NPs in the obese population[33, 34]. Bao et al. delved into the intricate interplay between BNP and blood lipids, aiming to enhance comprehension of the complex dynamics involving NPs and lipid levels. Their findings revealed an inverse relationship between NT-proBNP and LDL-C[35]. Similarly,

in a study by Spannella et al. conducted among an elderly population, a negative correlation was observed between levels of LDL-C and NT-proBNP, irrespective of whether NT-proBNP fell within the normal range[36]. This study showed a reduction in LDL-C levels after 16 weeks' Sacubitril/Valsartan medication compared to the pre-treatment phase. This observed decrease in LDL-C might be attributed to the elevated BNP content induced by Sacubitril/Valsartan, subsequently leading to LDL-C reduction. However, intergroup analysis revealed no significant disparity, prompting an analysis of the underlying reasons for this outcome. It was discovered that AngII induces LDL-C aggregation, thereby elevating the expression of LDLR. Remarkably, BNP inhibits AngII-induced LDLR expression, diminishing LDL-C binding and consequently lowering LDL-C levels[37, 38]. Moreover, Valsartan inhibits AngII binding to the receptor, which can also inhibit the metabolic processes of LDL-C. Therefore, these intricate interactions provided a plausible explanation for the study's negligible difference in LDL-C levels between groups. Following 16 weeks of treatment, ApoB decreased from baseline in both the Sacubitril/Valsartan and Valsartan group. This suggested that alterations in LDL-C may contribute to this observed phenomenon. Although TC levels diminished in both groups post-treatment, the lack of significant differences may be attributed to TC encompassing HDL-C and non-HDL-C, where even a slight alteration in each of these indicators could influence TC levels.

In light of the decrease in TG levels shown with Sacubitril/Valsartan treatment, the study conducted Spearman correlation analysis to unravel factors influencing TG reduction. Beyond the treatment modality, BMI and HbA1c emerged as significant

contributors to TG level changes. Individuals with both CKD and CHF exhibit a heightened prevalence of lipid abnormalities, a consequence of inflammatory factors, RAAS activation, and the interplay between heart and kidney functions. Notably, obesity, prevalent in the study's participants with a higher average BMI than normal adults, poses a risk factor for these participants. Therefore, regulating lipids and BMI become paramount in managing CKD and CHF patients. Oh et al.'s community study have demonstrated a positive relationship between elevated BMI and increased TG levels, highlighting the potential benefits of moderate weight management in reducing TG[39]. This study revealed a modest but positive correlation (r_s = 0.391) between declining TG levels and BMI. Nevertheless, this observation underscored a noteworthy reduction in TG levels among individuals with higher BMI following medication. Additionally, there was a correlation between the extent of TG reduction and HbA1c levels. Hsiung et al.'s Mendelian randomized study elucidated that elevated TG levels affect genomic methylation status, leading to increased blood HbA1c[40]. Zheng et al. demonstrated a close association between poor glycemic management and elevated TG levels in individuals with type 2 diabetes, emphasizing the independent contribution of elevated TG levels to suboptimal glycemic control, even in those with normal BMI. Hence, managing triglyceride levels might prove more efficacious in glycemic control[41]. This correlation underscored the importance of stringent lipid control, particularly in patients with high HbA1c levels, given the heightened risk of diabetic microvascular complications associated with elevated triglycerides[42].

On the other hand, numerous real-world clinical investigations have explored how

Sacubitril/Valsartan affects cardiac parameters in HF patients. By augmenting NP levels, inducing vasodilation, promoting sodium and urine excretion, and concurrently inhibiting the RAAS, the advantages of Sacubitril/Valsartan seem particularly pronounced in reducing heart failure mortality and reversing left atrial remodeling, especially among patients with a low LVEF[43, 44]. Within this study, featuring an intermediate ejection fraction type of heart failure, subjects that used Sacubitril/Valsartan manifested an obvious elevation in LVEF and decrease in LAD, in line with previous research. NT-proBNP holds significance in predicting heart failure prevalence, mortality, and prognosis[45], given that NT-proBNP is not an enkephalin substrate, this study opted for NT-proBNP analysis, excluding enkephalin degradation and providing a more accurate reflection of changes in ventricular wall pressure after Sacubitril/Valsartan treatment. Nevertheless, controversies persist regarding NT-proBNP alterations. A meta-analysis by Kang et al., encompassing 3460 patients, observed a significant reduction in NT-proBNP following Sacubitril/Valsartan treatment[46]. However, a double-blind randomized clinical trial comprising 335 heart failure patients, reported no difference in NT-proBNP reduction between Valsartan and Sacubitril/Valsartan treatments[47]. This study aligns with the latter, primarily due to the influence of age, liver and kidney function, infections, and other factors on NT-proBNP levels.

Sacubitril/Valsartan exerts its influence on the glomerular filtration rate by expanding the small incoming arterioles while constricting the small outgoing arterioles. Additionally, it enhances the activity of the NP system, fostering cardiac improvement

through the cGMP pathway, coupled with an elevation in renal perfusion[48]. Multicenter randomized trials have demonstrated Sacubitril/Valsartan's potential to diminish the risk of renal deterioration in people with HF, whether they had HFrEF or HFpEF[49]. However, outcomes from Haynes's HARP-III trial revealed that, after a 12-month course, the impact on renal function with Sacubitril/Valsartan was comparable to that of irbesartan[50]. In a separate 8-week investigation, Huang et al. reported a 22.0% incidence of renal function decline in HFrEF patients receiving Sacubitril/Valsartan[51]. In this study, although the Sacubitril/Valsartan group exhibited lower creatinine levels and higher eGFR levels post-treatment, no statistical distinction emerged between the two groups. The included individuals exhibited suboptimal average renal function, potentially accounting for this variation. The CKD population under scrutiny presented heightened hemodynamic alterations and inflammatory responses, and the observational period was relatively brief, preventing the manifestation of the enduring renal benefits of Sacubitril/Valsartan.

Concerning alterations in blood pressure, the study revealed a notable reduction in SBP among patients with CKD and CHF in intergroup comparisons. However, there was no significant change in DBP between the two groups. Prior research has consistently affirmed the effectiveness of Sacubitril/Valsartan in effectively lowering blood pressure, substantiating its utility in blood pressure management. Throughout the course of treatment, the potassium levels in both groups remained within the safe range, with no statistically significant differences.

Strengths and limitations of the study

This study presented the following advantages. First, the pioneering inclusion of patients grappling with both CKD and CHF established a crucial groundwork for lipid management, particularly in the context of employing Sacubitril/Valsartan within this specific demographic. Second, the focus of this inquiry on elucidating the influence of Sacubitril/Valsartan on lipid metabolism, in comparison to Valsartan, has introduced novel perspectives that may hold potential for broadening the scope of Sacubitril/Valsartan's utility in future scenarios.

However, this study was constrained by some limitations. First, patient data was obtained through the electronic medical record system, with the adjustment of medication doses for patients not consistently documented in real-time. The study duration was brief, the sample size limited, and post-16-week blood lipid status of patients was not monitored. Second, factors such as underlying patient conditions, irregular drug usage in treatments, and dietary alterations may influence the study outcomes, despite the absence of intergroup differences in baseline data. Third, the glomerular filtration rate of the included subjects was below 60 mL/min/1.73m^2, and renal impairment exerted a large effect on NT-proBNP, preventing a comprehensive examination of cardiac function alterations due to the inability to completely exclude the influence of renal factors. Therefore, the findings of the study necessitate exploration through broader, multicenter studies with larger sample sizes and extended prospective durations.

4 Conclusions

In comparison to Valsartan, Sacubitril/Valsartan demonstrates the capacity to diminish

levels of TG, elevates levels of HDL-C and ApoA in patients with CKD complicated with CHF, particularly demonstrating efficacy in TG reduction. Additionally, Sacubitril/Valsartan exhibits the potential to enhance cardiac function in patients without inducing notable deterioration of renal function. BMI and HbA1c emerge as influential factors for changes in TG levels, irrespective of Sacubitril/Valsartan. The promise of Sacubitril/Valsartan in modulating lipid metabolism is evident.

17805922_ManzhiLi.docx

ORIGINALITY REPORT

8%
SIMILARITY INDEX

PRIMARY SOURCES

1	"Abstracts Programme", European Journal of Heart Failure, 2019<br>Crossref	41 words — 1%
2	www.ncbi.nlm.nih.gov<br>Internet	36 words — 1%
3	www.science.gov<br>Internet	27 words — < 1%
4	Yin Ruixing, Chen Yuming, Pan Shangling, He Fengping et al. "Comparison of Lipid Levels, Hyperlipidemia Prevalence and Its Risk Factors between Guangxi Hei Yi Zhuang and Han Populations", Archives of Medical Research, 2006<br>Crossref	24 words — < 1%
5	Jingyi Ren, Jufeng Liang, Jiaqi Wang, Bowen Yin et al. "Vascular benefits of vitamin C supplementation against fine particulate air pollution in healthy adults: A double-blind randomised crossover trial", Ecotoxicology and Environmental Safety, 2022<br>Crossref	23 words — < 1%
6	Xia Zhong, Huachen Jiao, Dongsheng Zhao, Mengqi Yang, Jing Teng. "Association of Serum Apolipoprotein B Levels with Paroxysmal Atrial Fibrillation: A Case-Control Study", Research Square Platform LLC, 2022<br>Crossref Posted Content	23 words — < 1%

7 doctorpenguin.com
Internet
19 words — < 1%

8 Jaymi Anna George, Bhadrinath Srinivasan, Vignesh Kailasam. "The effect of active oxygen-containing toothpaste on Streptococcus mutans and white spot lesions: An in-vivo randomized controlled trial", American Journal of Orthodontics and Dentofacial Orthopedics, 2022
Crossref
18 words — < 1%

9 mdpi.com
Internet
16 words — < 1%

10 Sadia Mahmud Trisha, Sanjana Binte Ahmed, Md Fahim Uddin, Tahsin Tasneem Tabassum et al. "Prevalence, knowledge, causes, and practices of self-medication during the COVID-19 pandemic in Bangladesh: A cross-sectional survey", Cold Spring Harbor Laboratory, 2023
Crossref Posted Content
14 words — < 1%

11 reaganudall.org
Internet
14 words — < 1%

12 Po-Sheng Huang, Cheng-Hong Liu, Hsueh-Chih Chen. "Examining the applicability of representational change theory for remote associates problem-solving with eye movement evidence", Thinking Skills and Creativity, 2019
Crossref
13 words — < 1%

13 sci-hub.se
Internet
12 words — < 1%

14 Deniz Acar, Selahittin Çayan, Savaş Aktaş, Mesut Tek, Erdem Akbay. "The effect of tamoxifen on bladder functions and histology, and the role of estrogen
11 words — < 1%

receptor β in a rat chemical cystitis model", Neurourology and Urodynamics, 2007
Crossref

---

15 www.ajol.info
Internet
11 words — < 1%

---

16 kipdf.com
Internet
10 words — < 1%

---

17 mdpi-res.com
Internet
10 words — < 1%

---

18 pubmed.ncbi.nlm.nih.gov
Internet
10 words — < 1%

---

19 Moti L. Kashyap. "Optimal Therapy of Low Levels of High Density Lipoprotein-Cholesterol", American Journal of Cardiovascular Drugs, 2003
Crossref
9 words — < 1%

---

20 academic.oup.com
Internet
9 words — < 1%

---

21 fjfsdata01prod.blob.core.windows.net
Internet
9 words — < 1%

---

22 journal07.magtech.org.cn
Internet
9 words — < 1%

---

23 worldwidescience.org
Internet
9 words — < 1%

---

24 Ailin Liu, Na Sun, Feiyu Gao, Xiaotong Wang, Hong Zhu, Defeng Pan. "The prognostic value of dynamic changes in SII for the patients with STEMI undergoing PPCI", Research Square Platform LLC, 2023
Crossref Posted Content
8 words — < 1%

25	Catherine Coolens, Brandon Driscoll, Warren Foltz, Igor Svistoun, Noha Sinno, Caroline Chung. "Unified platform for multimodal voxel-based analysis to evaluate tumour perfusion and diffusion characteristics before and after radiation treatment evaluated in metastatic brain cancer", The British Journal of Radiology, 2019<br>Crossref	8 words — < 1%
26	Jing Xiong, Mingyu Qi, He Shi, Deli Zou, Quanxiang Liu, Wei Cheng. "Association between osteoporosis and coronary heart disease in the elderly", Research Square Platform LLC, 2023<br>Crossref Posted Content	8 words — < 1%
27	www.omicsdi.org<br>Internet	8 words — < 1%
28	www.researchsquare.com<br>Internet	8 words — < 1%
29	Joyita Banerjee, Neetu Mishra, Gauri Damle, Yogita Dhas. "Beyond LDL-c: The importance of serum oxidized LDL in predicting risk for type 2 diabetes in the middle-aged Asian Indians", Diabetes & Metabolic Syndrome: Clinical Research & Reviews, 2019<br>Crossref	7 words — < 1%

EXCLUDE QUOTES	ON	EXCLUDE SOURCES	OFF
EXCLUDE BIBLIOGRAPHY	ON	EXCLUDE MATCHES	OFF
